# *Bacillus firmus* I-1582 promotes plant growth and impairs infection and development of the cyst nematode *Heterodera schachtii* over two generations

**DOI:** 10.1038/s41598-021-93567-0

**Published:** 2021-07-08

**Authors:** Mengmeng Huang, Aylin Bulut, Bidhya Shrestha, Christiane Matera, Florian M. W. Grundler, A. Sylvia S. Schleker

**Affiliations:** INRES-Department of Molecular Phytomedicine, Rheinische Friedrich-Wilhelms-University of Bonn, 53115 Bonn, Germany

**Keywords:** Bacteria, Pathogens

## Abstract

Plant-parasitic nematodes wreak havoc on crops by root parasitism worldwide. An approach to combat nematode root parasitism is the application of antagonistic microbes like the rhizobacterium *Bacillus firmus* I-1582 which is promoted as biological control agent. Although *B. firmus* is a known nematode antagonist in general, the underlying mechanisms about its interaction with nematodes and plants have not yet been elucidated. Therefore, we explored the influence of *B. firmus* I-1582 as well as its extracellular and secreted molecules on plant–nematode interaction utilizing the plant–pathogen system *Arabidopsis thaliana*–*Heterodera schachtii*. We demonstrated that *B. firmus* I-1582 is attracted by *A. thaliana* root exudates, particularly by those of young plants. The bacterium colonized the root and showed a strictly pH-dependent development and plant growth promotion effect. Our results revealed that root colonization by *B. firmus* I-1582 significantly protected *A. thaliana* from infestation by the beet cyst nematode whereas dead bacterial cells or the culture supernatant were not effective. The bacterium also negatively affected nematode reproduction as well as pathogenicity and development of next generation nematodes. The obtained results highlight *B. firmus* I-1582 as a promising biocontrol agent that is well suited as an element of integrated control management strategies in sustainable agriculture.

## Introduction

Plant-parasitic nematodes (PPNs) cause severe losses in almost all kind of crops. PPNs mostly inhabit the soil and primarily attack underground parts of the plants. Therefore, it is often very difficult to diagnose, identify and control PPNs^1^. Nematode infestation affects plants on the one hand directly leading to growth reduction and lower crop yield, on the other hand, some PPN species are vectors of plant viruses or are associated with specific microbial root pathogens^2,3^. Over the past few decades, the most common approach of PPN management has been the application of synthetic nematicides. However, many synthetic products cause major safety concerns due to their general toxicity and their effects on non-target organisms, resulting in severe restrictions or complete banning^4^. Therefore, there is an urgent demand for the development and implementation of alternatives to control PPNs that provide effective and sustainable management while minimizing negative consequences for human health and environmental safety.

The rhizosphere is teeming with microscopic life forms including bacteria, fungi, and protozoa, thus creating possibilities of using specific organisms as biocontrol agents against soil-borne pathogens^5–7^. Among the range of soil organisms, the plant growth-promoting rhizobacteria (PGPR) were found to have promising potentials. PGPR are bacteria which have the ability to colonize the roots thereby stimulating plant growth and/or suppressing plant diseases^8,9^. For example, the application of *Pseudomonas* strains has shown the favourable effect of increasing yield of potato, sugar beet, and radish^10–12^. Certain *Bacillus* strains have also been announced to have antagonistic activity against soil-borne pathogens^6,13–15^. According to the mode of action, PGPR are divided into two groups that show either direct or indirect mechanisms. Direct contribution includes secreting phytohormones, facilitating nutrition acquisition, and producing volatile organic compounds, while indirect benefit consists of restraining deleterious rhizosphere microorganisms through antibiosis, competition for space and nutrients, and parasitism of pathogenic agents, and/or inducing systemic resistance^16–18^.

Previous studies have found that PGPR usually require efficient root surface colonization to exert their beneficial effects on plants^9,19,20^. However, to achieve successful colonization, chemotaxis of PGPR towards the root system is considered as a prerequisite^21,22^. Chemotaxis enables bacteria to sense a wide range of signals, and guides them to an appropriate environment for survival. Accumulative evidence suggests that root exudates are active in initiating and regulating this chemotactic response in the rhizosphere^23–26^. Root exudates, consisting of amino acids, organic acids (OAs), sugars, phenolics, polysaccharides, and proteins, provide nutrition for PGPR and act as signals to attract or repel microorganisms^27,28^. The composition of root exudates may vary over the course of plant development. For example, secretion of sugars from *A. thaliana* roots reaches the greatest abundance at an early development stage, while the quantity of secreted amino acids and OAs increases during development^29^.

*Bacillus firmus*, which was first reported in 1933, is an aerobic, alkaliphilic, Gram-positive, and endospore-forming bacterium^30^. *B. firmus* has been shown to possess a great potential in promoting the growth of host plants, such as tomato, cotton, and Bermudagrass^31–33^. It has also been well characterized in a series of laboratory, greenhouse and field studies for its nematicidal property against a wide range of nematodes, such as the root-knot nematode *Meloidogyne incognita*^33–36^, the soybean cyst nematode *Heterodera glycines*^37,38^, the burrowing nematode *Radopholus similis*^34,39^, and the stem nematode *Ditylenchus dipsaci*^34^. Greenhouse experiments demonstrated that the application of *B. firmus* on tomato plants not only efficiently reduce gall index, egg masses and final populations of *M. incognita*, but also significantly increase plant height, plant biomass, fruit number, and fruit weight^33,36,40^.

The strain *Bacillus firmus* I-1582 was originally isolated from soil obtained from the central plain area in Israel. *B. firmus* I-1582 is easy to formulate and is commercially available as a seed treatment or as a wettable powder for the bio-control of PPNs^41^. The spores are able to persist durably in fields. Despite its appealing traits, the effects accomplished by *B. firmus* I-1582 is often unpredictable. The main reason for this is a lack of knowledge of the complex biology of the control system. Deeper understanding of *B. firmus* I-1582 and its overall interactions with nematodes and plants is a pre-requisite for improving biocontrol.

In the present study, we approached this by (i) examining the attractiveness of *A. thaliana* root exudates for *B. firmus* I-1582, (ii) determining the optimal pH for *B. firmus* I-1582 colonization of *A. thaliana* roots and its impact on plant development; (iii) establishing a robust ternary in vitro test system for the evaluation of nematode parasitism at *A. thaliana* in the presence of *B. firmus* I-1582. Utilizing this sophisticated agar-based gnotobiotic system enabled us to analyse the plant-promoting effect and nematode-antagonistic activity of *B. firmus* I-1582, and facilitated us to explore these interspecies interactions in detail.

## Results

### Chemotactic response of *B. firmus* I-1582 towards *A. thaliana* root exudates and components

To understand the initiation of root colonization by *B. firmus* I-1582, its chemotactic response to root exudates of *A. thaliana* Col-0 was investigated. It has been reported that bacteria are more motile and more strongly chemotactic in the late exponential phase^42^. Thus, we first determined *B. firmus* I-1582 development under defined growth conditions. In order to standardize the procedure, the main culture was always inoculated with a fresh overnight culture to obtain an initial OD_600_ of 0.1 (Suppl. Fig. 1). After an initial lag phase of about 2 h, the growth rate increased sharply between 2.5 and 5 h, and began to taper off until reaching stationary phase at about an OD_600_ of 3. Accordingly, the OD_600_ between 2.0 and 2.5 was determined as *B. firmus* I-1582 late exponential phase, and chemotaxis assays were performed using bacteria in this growth phase.

**Figure 1:**
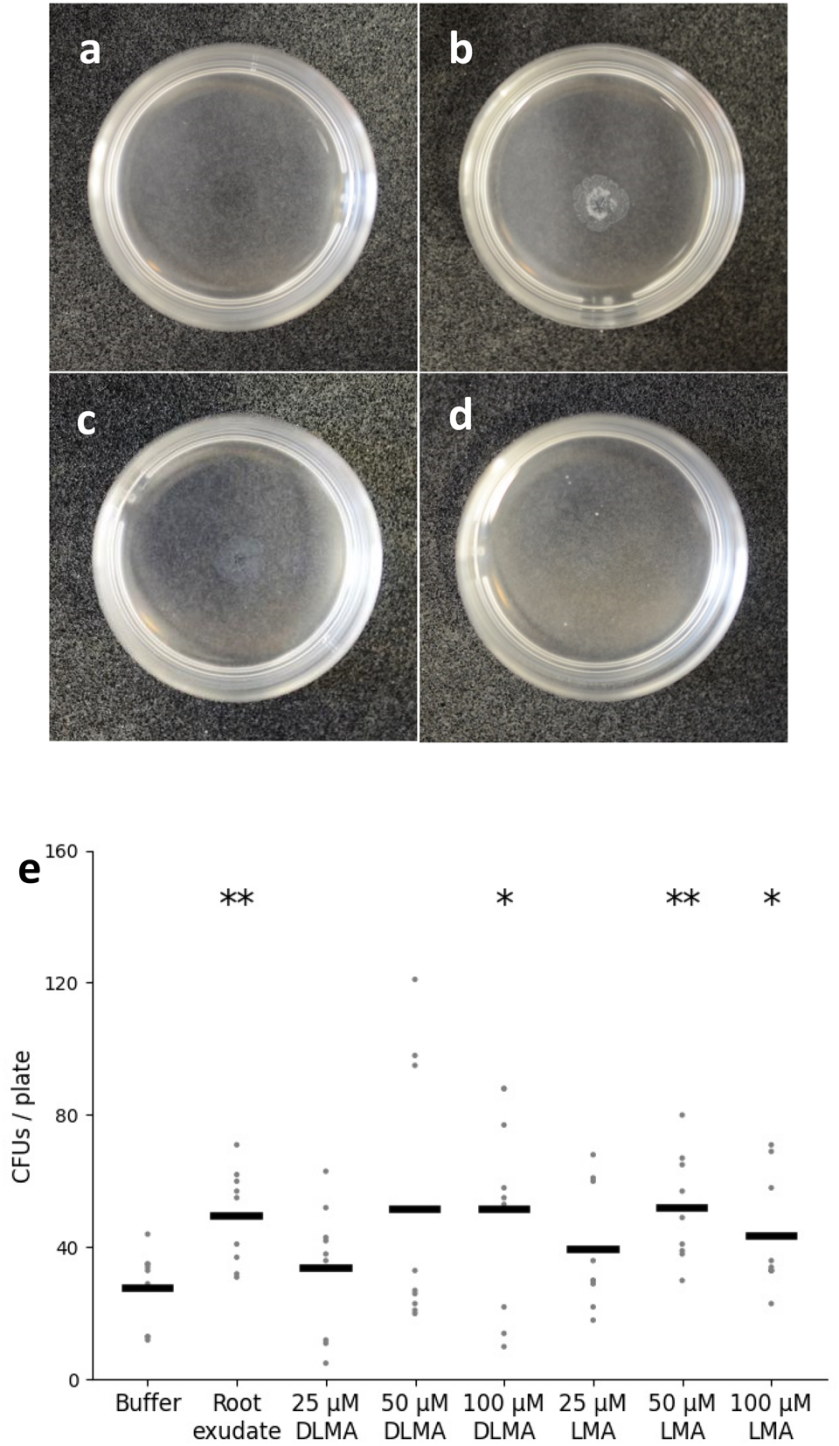
Chemotactic response of *Bacillus firmus* I-1582 towards *Arabidopsis thaliana* root exudates (AREs) (**a**–**e**) and organic acids (OAs) (**e**). Upper row: A drop of the test substance was placed in the centre of a 35 mm diameter Petri dish containing *B. firmus* I-1582. A turbid ring indicated the aggregation of bacteria. 10 µl of chemotaxis buffer (A); 10 µl of AREs extracted from 7- (**b**), 21- (**c**), and 28- (**d**) day-old *A. thaliana* plants. Photographs are representative examples taken after incubation for 30 s at room temperature. Bottom graph (**e**): Chemotactic response of *B. firmus* I-1582 towards AREs of 7-day-old seedlings and OAs in a capillary assay. *DLMA* dl-malic acid, *LMA* l-malic acid, *AREs* *A. thaliana* root exudates, *OAs* organic acids. Results are expressed as the mean ± standard error of three independent biological replicates (n = 9). Data are statistically analysed using Student’s *t* test. Asterisk (*) indicate statistically significant differences compared with the control (*p < 0.05, **p < 0.01).

In order to obtain a qualitative indication whether and how effective the bacterium is attracted by root exudates, *B. firmus* I-1582 was exposed to *A. thaliana* root exudates (AREs) harvested from plants of three different developmental stages (Fig. 1a–d, Suppl. Fig. 2). The turbid ring surrounding the centre of the Petri dish indicated that bacterial cells aggregated around the concentrated AREs placed there. In contrast to the control, the size of the chemotactic ring increased with increasing volume of root exudate. Moreover, bacterial cells were attracted to all the AREs of plants from different development stages. The strongest chemotactic ring appeared when using AREs from 7-day-old plants. This qualitative test showed that *B. firmus* I-1582 is attracted by *A. thaliana* root exudates, particularly by those of young plants.

Subsequently, the chemotactic response of *B. firmus* I-1582 towards AREs freshly extracted from 7-day-old *A. thaliana* plants, and malic acid previously identified in the AREs was validated in a quantitative assay^43^. The concentrated AREs as well as dl-malic acid and l-malic acid at 50 and/or 100 µM caused a positive chemotactic response to *B. firmus* I-1582 (Fig. 1e). Thus, the capillary assay further supports the observations of the qualitative drop assay.

### Establishment of a ternary in vitro test system

Since *B. firmus* I-1582 is an alkaliphilic bacterium, the pH of the surrounding environment might be a crucial factor for successful root colonization and bacterial development. Therefore, the behaviour of *B. firmus* I-1582, *H. schachtii*, and *A. thaliana* at different pH levels was studied.

**Figure 2:**
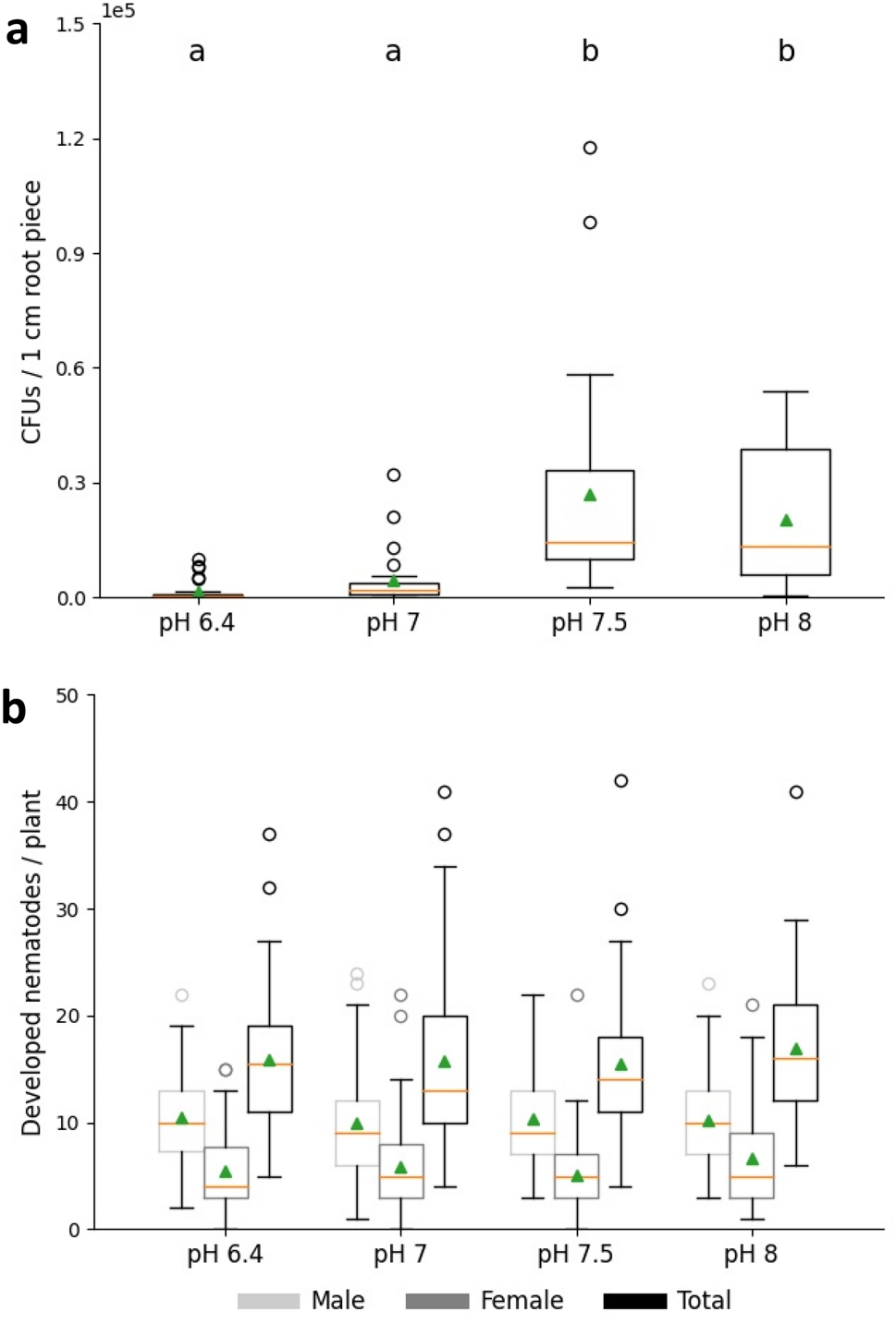
Impact of pH on colonization of *Bacillus firmus* I-1582 (**a**) and infection of *Heterodera schachtii* (**b**) at *Arabidopsis thaliana* roots. Results are expressed as the mean (orange line) and median (green triangle) ± standard error of three independent biological replicates [n ≥ 22 (**a**); n ≥ 50 (**b**)]. Different letters indicate statistically significant differences among treatments according to Dunn’s Method (*p* < 0.05). No significant differences were observed for (**b**).

We observed that attachment and development of *B. firmus* I-1582 at *A. thaliana* roots are strongly pH-dependent (Fig. 2a). Twelve days after inoculation, 1.7 × 10^3^ CFUs and 4.4 × 10^3^ CFUs per 1 cm root piece were detected at pH 6.4 and pH 7, respectively. Bacterial counts significantly increased by 80–90% to 2.7 × 10^4^ and 2.0 × 10^4^ CFUs at pH 7.5 and pH 8, respectively.

As pH has a tremendous impact on *B. firmus* development at the root, it was analysed whether parasitic success of *H. schachtii* at *A. thaliana* is influenced by the pH levels applied. It was confirmed that there was no significant difference in the average number of developed females, males, and total nematodes as well as the average size of females at the four different medium pH values (Fig. 2b and Suppl. Fig. 3). To avoid overgrowth of bacteria in the test system, the growth medium did not contain sucrose. Based on Hofmann et al.^44^, limited carbohydrate supply can affect sexual differentiation leading to a reduced number of females and a shift towards male development. This can also be observed in our experiments.

**Figure 3:**
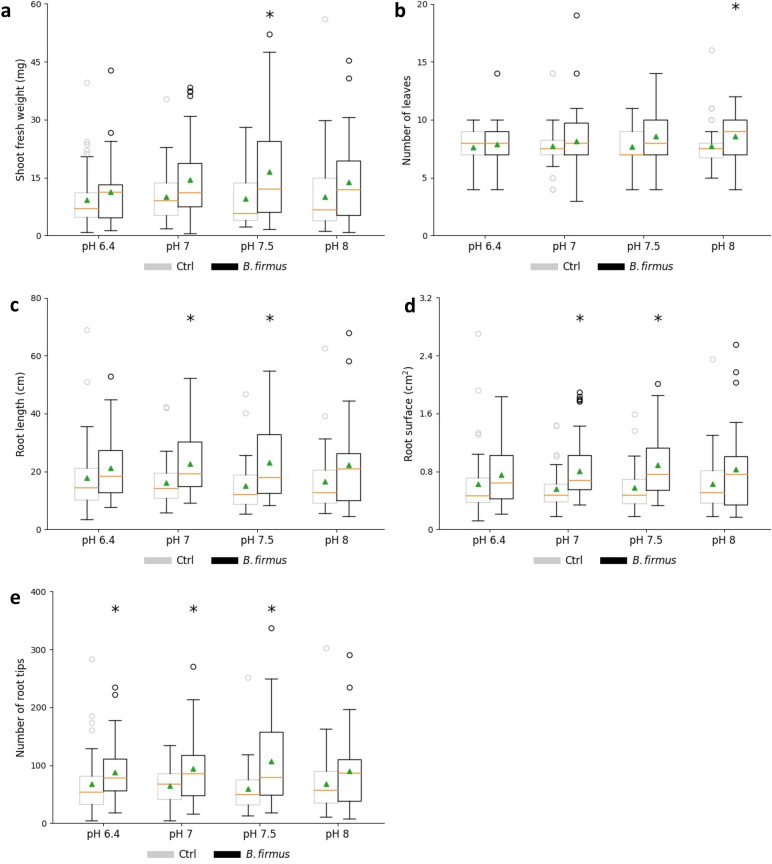
Impact of pH and *Bacillus firmus* I-1582 inoculation on *Arabidopsis thaliana* development. Average shoot fresh weight (**a**), average number of leaves (**b**), average total root length (**c**), average root surface (**d**), average number of root tips (**e**) per plant. Results are expressed as the mean (orange line) and median (green triangle) ± standard error of three independent biological replicates (n ≥ 31). *Ctr* control. Data are statistically analysed using Dunn’s Method. Asterisk (*) indicate statistically significant differences compared with the control (*p* < 0.05).

Next, we investigated the impact of pH and the bacterium on plant phenotype (Fig. 3). Plants without bacterial treatment grown at different pH exhibited no differences for all aboveground and belowground parameters. Inoculation with *B. firmus* I-1582 resulted in a significant increase of shoot fresh weight at pH 7.5. The leaf number also showed an increasing trend when the root is exposed to the bacterium. All belowground parameters (root length, root surface and root tips) of the variants with bacterium at pH 7 and pH 7.5 had significantly higher values compared with the bacteria-free ones. Taken together, pH 7.5 is the optimal pH of the growth medium for testing the impact of *B. firmus* on *H. schachtii* in our gnotobiotic *A. thaliana* test system.

### Impact of *B. firmus* I-1582 on *H. schachtii* parasitism at *A. thaliana*

**Figure 4:**
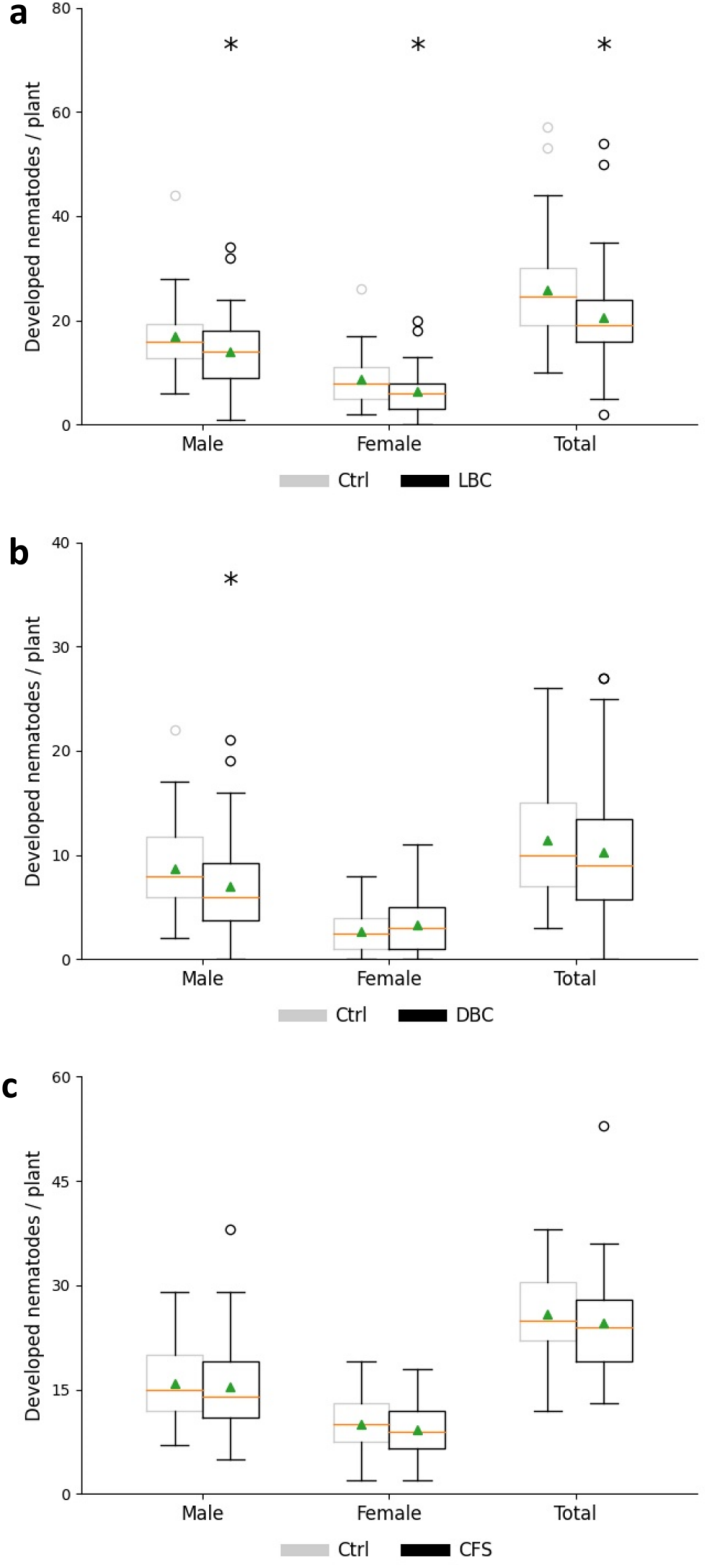
Infection assay of *Heterodera schachtii* at *Arabidopsis thaliana* roots. Average number of nematodes per plant at 14 dpi (days post inoculation) in the presence of LBC (**a**), DBC (**b**) or CFS (**c**). *LBC* living bacterial cells, *DBC* dead bacterial cells, *CFS* cell-free supernatant. Results are expressed as the mean (orange line) and median (green triangle) ± standard error of three independent biological replicates (n ≥ 40). Data are statistically analysed using Dunn’s Method. Asterisk (*) indicate statistically significant differences compared with the control (*p* < 0.05).

In order to determine nematode susceptibility to *B. firmus* I-1582 and to get an indication for the mechanism, infection assays were performed in the presence of living bacterial cells (LBC), dead bacterial cells (DBC) or cell-free supernatant (CFS). Three parameters (nematode number, female size and syncytium size) indicating the parasitic success of nematodes were evaluated. Treatment with LBC, DBC or CFS was differently efficient against *H. schachtii*. The presence of living cells caused a significant reduction of the number of developed males, females and total nematodes at 14 dpi, with an average decrease of 17.7%, 26%, and 20.6% compared with the control, respectively (Fig. 4a). Inoculation with DBC only reduced male number by 19.1%, while CFS did not have any suppressive impact on nematode parasitism (Fig. 4b,c).

**Figure 5:**
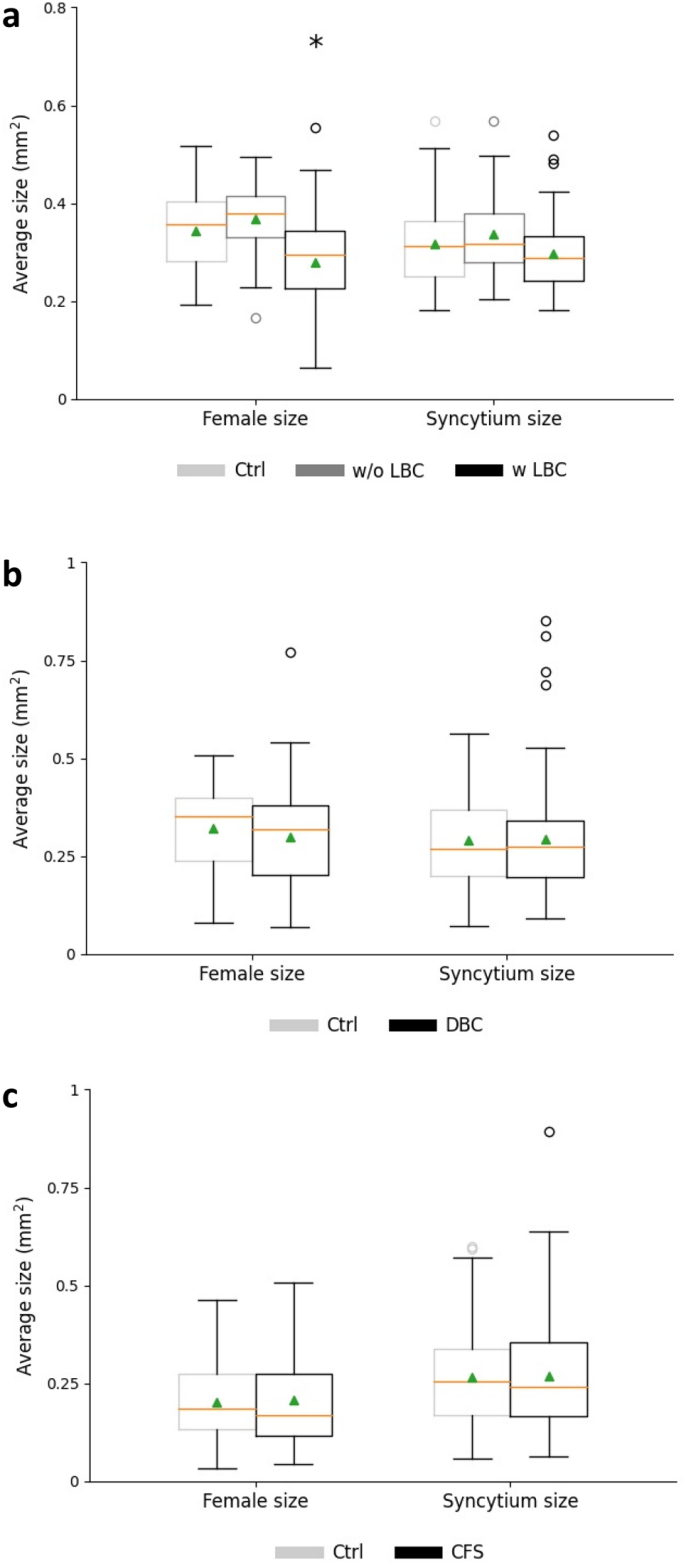
Development of *Heterodera schachtii* at *Arabidopsis thaliana* roots. Average size of female and syncytium at 28 dpi (days post inoculation) in the presence of LBC (**a**), DBC (**b**) or CFS (**c**). *LBC* living bacterial cells, *DBC* dead bacterial cells, *CFS* cell-free supernatant. Results are expressed as the mean (orange line) and median (green triangle) ± standard error of three independent biological replicates (n ≥ 53). Data are statistically analysed using Dunn’s Method. Asterisk (*) indicate statistically significant differences compared with the control (*p* < 0.05).

In addition, we found that the direct contact of the female nematode to living bacteria is a key factor to stunt nematode development. At 28 dpi, the females surrounded by bacteria (w LBC) were significantly smaller in size (18.6%) compared with those of the control, while the females developed at the bacterium-treated plants that were not colonized by bacteria (w/o LBC) were not different in size compared with the females at the control plants (Fig. 5a). DBC and CFS treatment did not influence female development (Fig. 5b,c). None of the treatments had an impact on the female-associated feeding site (Fig. 5).

**Figure 6:**
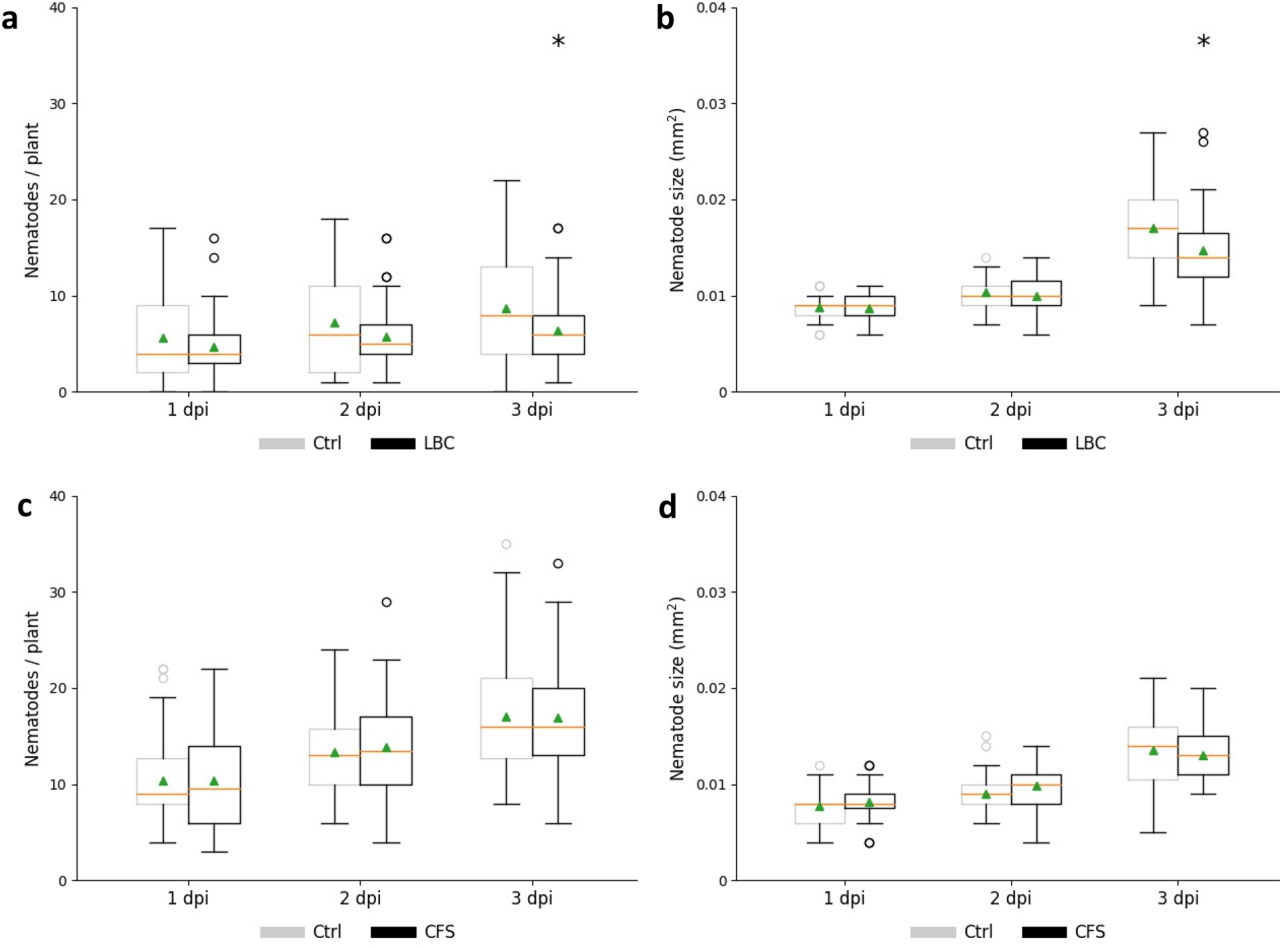
Invasion assay of *Heterodera schachtii* at *Arabidopsis thaliana* roots. Average number of nematodes per plant in the presence of LBC (**a**) or CFS (**c**); Average size of nematodes in the presence of LBC (**b**) or CFS (**d**). *LBC* living bacterial cells, *CFS* cell-free supernatant, *dpi* days post inoculation. Results are expressed as the mean (orange line) and median (green triangle) ± standard error of three independent biological replicates (n ≥ 37). Data are statistically analysed using Dunn’s or Holm–Sidak Method. Asterisk (*) indicate statistically significant differences compared with the control (*p* < 0.05).

Since *B. firmus* I-1582 living cells had a considerable impact on *H. schachtii* parasitism, we decided to study nematode interaction with plant and bacteria in more detail. For this purpose, the early events after nematode inoculation were investigated. It was observed that *H. schachtii* started to invade the root at 1 dpi but penetrated at a decreased rate at 1, 2 and 3 dpi when exposed to LBC (Fig. 6a). In addition, at 3 dpi nematode size was significantly reduced in the bacteria-treated variant (13.8%) compared with the control (Fig. 6b). As expected, CFS treatment neither influenced nematode penetration nor nematode development (Fig. 6c,d).

### Impact of *B. firmus* I-1582 on *H. schachtii* progeny

As *B. firmus* I-1582 living cells play a vital role in preventing nematode parasitism at *A. thaliana* roots, the influence of *B. firmus* application on the amount and infectivity of the next generation juveniles as well as their development at the host plant was investigated. Therefore, cysts from bacteria-inoculated plants were separated into two groups: those with microscopically visual attachment of bacterial (w LBC) and those obviously not in contact with bacteria (w/o LBC). Cysts from plates without bacterial inoculation served as control. Juveniles hatching from cysts of the 3 different groups were used to inoculate *A. thaliana* and to investigate parasitic parameters. The cysts that developed at the end of these 2nd generation infection assays were analysed for presence of bacteria. Although the attachment of bacteria in the ‘w/o LBC’ group of the 1st generation cysts was not visible under the microscope, the amount of bacteria attached to the 2nd generation cysts was quantifiable (Suppl. Fig. 5). On average 8 and 5 CFUs could be determined on the 2nd generation cysts that developed from juveniles that hatched from the 1st generation cysts of the ‘w LBC’ group and the ‘w/o LBC’ group, respectively. Thus, we concluded that the two groups represent 1st generation cysts with a different bacterial load and are subsequently referred to as ‘w hLBC’ (with high number of LBC) and ‘w lLBC’ (with low number of LBC).

**Figure 7:**
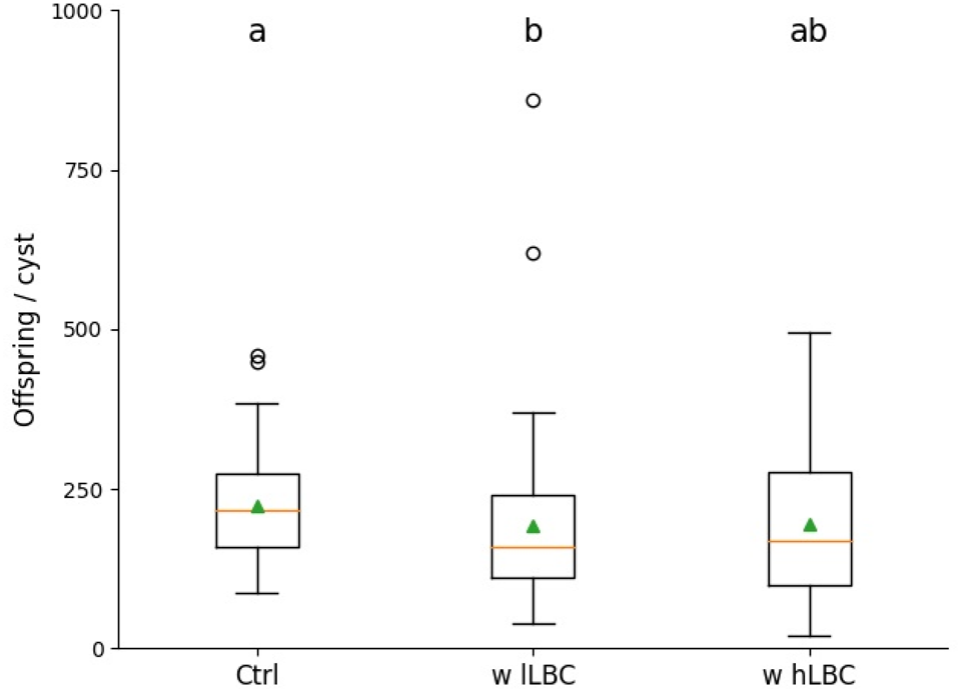
Reproduction assay of *Heterodera schachtii* at *Arabidopsis thaliana* roots. Average number of eggs and juveniles per cyst at 35 dpi in the presence of LBC. *w hLBC* with high number of living bacterial cells, *w lLBC* with low number of living bacterial cells. Results are expressed as the mean (orange line) and median (green triangle) ± standard error of three independent biological replicates (n ≥ 49). Different letters indicate statistically significant differences among treatments according to Dunn’s Method (*p* < 0.05).

Egg counts revealed that *B. firmus* reduced the number of offspring. First generation cysts of the ‘w lLBC’ group harboured a significantly lower number of offspring in comparison to those of the untreated control. The average egg numbers of cysts of the ‘w hLBC’ group was considerably lower compared with the control though not significantly different to the control or the ‘w lLBC’ group (Fig. 7). The CFS treatment had no influence on nematode reproduction (Suppl. Fig. 6).

**Figure 8:**
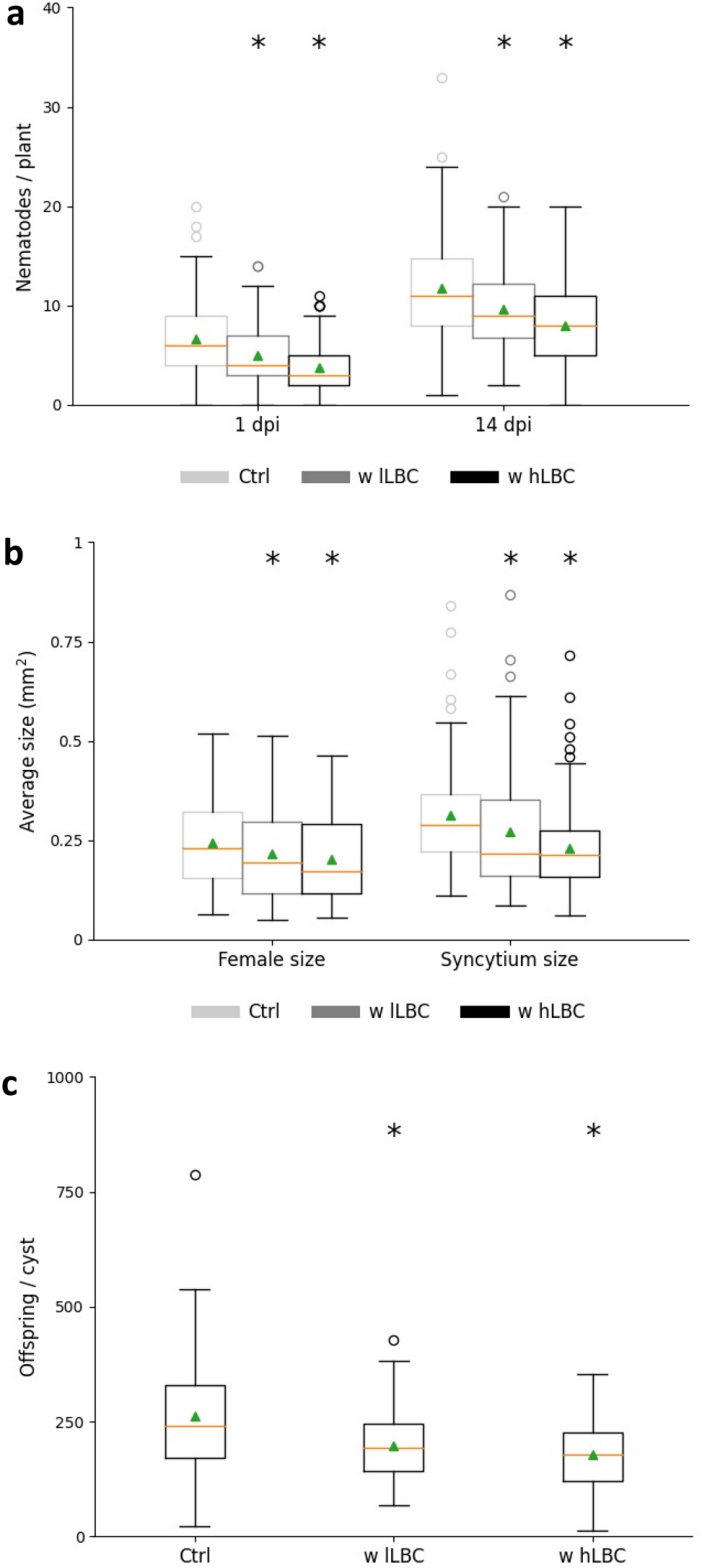
*Arabidopsis thaliana* root parasitism of *Heterodera schachtii* 2nd generation nematodes originating from *Bacillus firmus* colonized cysts. Average number of nematodes per plant at 1 dpi and 14 dpi (**a**); Average size of female and syncytium at 28 dpi (**b**); Average number of eggs and juveniles per cyst at 35 dpi (**c**). *w hLBC* with high number of living bacterial cells; *w lLBC* with low number of living bacterial cells; *dpi* days post inoculation Results are expressed as the mean (orange line) and median (green triangle) ± standard error of three independent biological replicates (n ≥ 97). Data are statistically analysed using Dunn’s Method. Asterisk (*) indicate statistically significant differences compared with the control (*p* < 0.05).

The virulence of hatched juveniles from control cysts and those with bacterial colonization was assessed by evaluating nematode invasion, infection and reproduction. As illustrated in Fig. 8 A, nematode penetration number (at 1 dpi) and adult number (at 14 dpi) were significantly decreased by 25.5% and 17.8%, respectively for the ‘w lLBC’ variant, while higher bacterial load caused a larger reduction of nematode invasion and establishment by 43.4% and 31.5%, respectively. The development of females and syncytia was also significantly suppressed by high (17.7% and 26.4%, respectively) or low (11.5% and 13%, respectively) bacterial amounts on the cysts of the previous generation (Fig. 8b). Second generation cysts originating from juveniles of the ‘w lLBC’ and the ‘w hLBC’ 1st generation cyst groups contained significantly fewer eggs compared with those of the untreated control group, with a reduction of 24.7% and 32.2%, respectively (Fig. 8c).

## Discussion

PGPR are commercially applied for biofertilization, phytoremediation, phytostimulation or biocontrol of soil-borne plant diseases^17^. Gaining insights into the interaction between plant and PGPR will contribute to improve effective application of these organisms in agriculture. Here, *B. firmus* I-1582 is investigated for its associations with *A. thaliana* and its efficiency to combat *H. schachtii* over 2 nematode generations.

Successful colonization of the root system by PGPR is an important factor for effective biological control^45^. Prior to root colonization, chemotaxis of PGPR towards root exudates is regarded to be a “sine qua non”^21^. Zhang et al.^46^ reported that *Bacillus subtilis* N11 was able to colonize banana root and prevent fungal infection by forming biofilms along the root. Subsequently, it was proved that the tested strain, isolated from banana rhizosphere, showed a stronger chemotactic response towards banana root exudates than to cucumber root exudates^47^. We observed that *B. firmus* I-1582 is attracted by AREs from differently aged *A. thaliana* plants. GC–MS analysis pointed out that AREs released from early development stage contain high level of sugars^29^. A high level of available carbon sources might be an explanation why we observe the strongest chemotactic response of *B. firmus* I-1582 to AREs extracted from young plants. During aging plants release a higher proportion of amino acids and phenolics resulting in a higher antimicrobial activity^48^. In fact, our data confirmed that the accumulation of bacterial cells was lowered by using AREs from older plants. Besides, an attraction assay was carried out utilizing AREs and two OAs. Result indicated that the chemotactic mobility of *B. firmus* I-1582 to dl-malic acid and l-malic acid were comparable to AREs. dl-Malic acid and l-malic acid are known to effectively recruit *B. subtilis* and stimulate biofilm formation of *B. subtilis* via a KinD-Spo0A pathway^43,49^. Based on our study, malic acid from AREs in fact seems to play an essential role in the chemoattraction of *B. firmus* I-1582.

Plenty of evidence demonstrated the antagonistic action of *B. firmus* species towards plant-parasitic nematodes. However, there is only little information about its interaction with and morphological impact on host plants. Consequently, the role bacteria–plant interaction plays in combating nematode infestation is not well studied but is essential as it may help us understand the interplay of the three organisms: the host plant, *B. firmus* and PPNs. Since *Bacillus* species are alkaliphiles^50^, we expected that colonization of *B. firmus* I-1582 at *A. thaliana* roots is pH-dependent. In fact, propagation of *B. firmus* I-1582 was remarkably enhanced in plant-growth medium at pH 7.5 and 8. In accordance, the tested strain also had a positive impact on the growth of *A. thaliana* in a pH-dependent manner. Shoot weight was significantly increased at pH 7.5. The belowground biometrical parameters of *A. thaliana* significantly increased after inoculation with *B. firmus* I-1582 in our axenic system at pH 7 and 7.5. In particular, attention should be payed to the expansion of the root system mirrored by the total root length, the root surface area and the number of root tips. An increase in root surface area indicates an increase of root hair formation. Root hairs facilitate plants in nutrient uptake and microbe interactions^51^.

PGPR can promote plant growth by biosynthesis of phytohormones^52^. Indole-3-acetic acid (IAA) secreted by *Bacillus amyloliquefaciens* UCMB5113 has been reported for its ability to induce *A. thaliana* plant cell division, and stimulate the development of lateral roots and root hairs^53^. Some other *Bacillus* strains, such as *Bacillus cereus*, *Bacillus flexus*, *Bacillus licheniformis*, *Bacillus megaterium*, *B. subtilis*, and *Bacillus thuringiensis*, were also able to synthesize IAA and could be considered as possible growth promoters of plant^54–57^. In addition, the increase of aboveground parameters of treated plants could be attributed to volatile organic compounds (VOCs) produced by PGPR strains^18^. The extracted bacterial volatiles from two *Bacillus* strains, *B. subtilis* GB03, *B. amyloliquefaciens* IN937a, significantly increase total leaf surface area of *A. thaliana*^58^. 2,3-Butanediol, a bacterial compound detected from volatile blends by GC analysis, was found to be released from both *Bacillus* strains and involved in the cytokinin-signaling pathway to activate plant growth. Regarding the biometrical response of *A. thaliana* seedlings, *B. firmus* I-1582 most probably impacts plant growth by releasing IAA and/or VOCs.

In our study, we validated that *H. schachtii* is susceptible to the presence of LBC as the bacterium significantly reduced the number of developed females and males, female size, and reproduction. Additionally, we proved that the LBC protects the host plant from J2 invasion and interferes with the nematodes’ development at the early sedentary phase. In contrast, the CFS as well as the DBC was not effective. Possible explanations for the observed characteristics are as follows.

- Microscopic observations during the reported study showed that the bacterium colonizes the root system building layers of bacterial cells along the root. This is in accordance with a recent study documenting *B. firmus* biofilm formation and colonization of tomato and cucumber roots^59^. Thus, it might be that the bacterium constitutes a physical barrier protecting the plant from nematode invasion.
- Generally, PPNs locate their preferred host by perceiving root exudate signals^60^. Strigolactones, which are a class of phytohormones exuded by Arabidopsis roots, were proven to play an active role in host attraction during beet cyst nematode parasitism^61^. Moreover, AREs were demonstrated to stimulate stylet thrusting of PPNs^62,63^. Simultaneously, root exudates also act as an attractant to and/or a rich source of nutrients for rhizobacteria^64,65^. Thus, it might be that the presence of *B. firmus* changes composition of root ARE in a way that roots become less attractive to the nematodes thus leading to a lower number of infection events.
- *Bacillus sphaericus* strain B43 was shown to stimulate induced systemic resistance against potato cyst nematode *Globodera pallida* juveniles in a split-root-trial^66^. *B. firmus* I-1582 is reported to induce systemic resistance in tomato active against *M. incognita* but not in cucumber^58^. Moreover, *Bacillus pumilus* T4 was capable to induce resistance in transgenic NahG Arabidopsis against two *Pseudomonas syringae* strains by activating SA-independent pathway^67^. Therefore, one possibility is that *B. firmus* I-1582 elicits plant defence responses effective against *H. schachtii*.
- There is a possibility that molecules secreted by *B. firmus* I-1582 are directly antagonistic against PPNs. According to Geng et al.^68^, *B. firmus* DS-1 isolated from marine sediment of the coast of the South China Sea has toxicity against *M. incognita* and *H. glycines*. They unravelled that a novel nematicidal virulence factor, Serine Protease 1 (Sep1), plays a role in nematode control by its serine protease activity. Sep1 was shown to target and degrade intestinal and cuticular proteins of *M. incognita*. In our study, we observed that CFS is not effective in inhibiting *H. schachtii* parasitism. Therefore, it is possible that the concentration of putative molecules involved in the nematicidal activity is too low in the CFS or/and that these virulence factors are secreted upon contact with the nematode.
- Based on our finding, virulence of the 2nd generation nematodes towards the host plant was decreased after exposure of 1st generation females to *B. firmus* I-1582 living cells. Since there are no bacteria established at the root, invasion of the 2nd generation juveniles cannot be influenced by a bacterial barrier along the roots or behavioural changes due to an altered root exudate composition. Thus, *B. firmus* I-1582 might reduce fitness of the 2nd generation juveniles before they hatch or bacterial attachment to the 2nd generation J2s directly influences the nematodes’ virulence. Alternatively/additionally, the J2-attached bacteria could trigger plant defence responses effective against the nematode.

Studies usually only consider the impact of an agent on one generation of nematodes on the host. However, *H. schachtii*—like other PPNs—has several generations within one vegetation period. Additionally, the juveniles remain viable inside the cysts for several years ready to hatch once the soil reaches appropriate temperature and moisture, and a suitable host plant is present. Therefore, we also examined the impact of *B. firmus* I-1582 living cells on the virulence of *H. schachtii* progeny inside the bacteria-colonized cysts. Intriguingly, J2s hatching from these cysts were significantly impaired in their development and reproduction at the host plant. The bacterial load of the developing 2nd generation cysts was with an average of about 8 CFU per cyst fairly low. Our results demonstrate that a single root application of *B. firmus* I-1582 not only reduces the plant’s infestation with the attacking nematode and the nematode’s reproduction but is also efficient in suppressing infection, development, and reproduction of the emerging 2nd nematode generation on a new host plant that was not treated with *B. firmus* I-1582 before. Future studies will reveal how strong the effects of *B. firmus* I-1582 against *H. schachtii* that we observed in our controlled in vitro studies will be in pot and field experiments. In conclusion, *B. firmus* I-1582 is a biological nematode control agent with high potential and sustainability that can be worth to integrate in PPN management strategies.

## Materials and methods

### Bacterial strain and culture conditions

*B. firmus* I-1582 was obtained from Bayer AG, Monheim and routinely stored in Tryptic soy broth (TSB) with 25% glycerol at − 80 °C. The strain was grown at 28 °C in liquid TSB with an overnight orbital shaking at 200 rpm. The main culture was inoculated with the overnight culture to obtain an initial optical density at 600 nm (OD_600_) of 0.1. The main culture was grown at 28 °C at 200 rpm for different time periods depending on the subsequent applications.

### Plant material and growth conditions

*A. thaliana* Columbia (Col-0) seeds were first surface-sterilized in 0.7% sodium hypochlorite for 5 min, then submerged in 70% ethanol for 1 min, and finally rinsed with sterile distilled water 5 times. Subsequently, the seeds were dried at room temperature for 4 h and stored at 4 °C until use. Surface-sterilized seeds were germinated in either normal Knop medium at pH 6.4 or modified Knop medium as described in the relevant sections, and kept in a climate chamber under a red/blue light with a 16-h/8-h light/dark photoperiod at 24 °C^69^.

All experiments involving plants were performed with *A. thaliana* Col-0. *Sinapis alba* was used for nematode propagation. All local, national or international guidelines and legislation were adhered to in the study.

### Nematode preparation

Approximately 300 cysts of *H. schachtii* were harvested from mustard (*Sinapis alba*) roots grown aseptically, and submerged with sterile 3 mM ZnCl_2_ in the Baermann funnel. After 7 days, the freshly hatched second-stage juveniles (J2s) were collected for subsequent analyses^61^.

### Chemotactic response of *B. firmus* I-1582 towards *A. thaliana* root exudates

#### Bacteria preparation

The OD_600_ of the main culture was measured every 30 min in triplicate to determine the growth rate of *B. firmus* I-1582 strain. Bacterial cells which reached the late exponential growth phase (OD_600_ of 2.0–2.5) were harvested by centrifugation [Eppendorf, Germany] at 4000 rpm for 10 min, washed with chemotaxis buffer (100 mM potassium phosphate [pH 7.0], 20 µM EDTA), and re-suspended in 16 ml of chemotaxis solution (12 ml of chemotaxis buffer and 4 ml of 1% hydroxypropylmethylcellulose). The resulting cell suspension was applied to two chemotaxis assays immediately after preparation.

#### Root exudates collection

AREs were collected from 7-, 21-, and 28-day-old seedlings. Seedlings were washed with sterile water, and incubated in sterile water for 3 days with orbital shaking at 100 rpm in a climate chamber under a red/blue light with a 16-h/8-h light/dark photoperiod at 24 °C. The collected AREs were filtrated through a 0.2 µm pore size syringe filter, and concentrated by lyophilisation [Thermo Scientific, USA] at − 104 °C until the volume reduced to 0.5 ml. The resulting AREs were stored at − 80 °C until further analyses.

#### Chemotaxis assays

The chemotactic response of *B. firmus* I-1582 towards the chemo-attractants (AREs and individual exudate components) was qualitatively examined by drop assay and quantitatively evaluated by capillary assay.

In the drop assay, the concentrated AREs (10 µl and 20 µl), which were collected from different development stages (7-, 21-, and 28-day), were dropped into the centre of a 35-mm-diameter Petri dish which contained 2 ml of bacterial cell suspension. A ring of turbidity near the centre of each petri dish would appear after 30 s of incubation at room temperature, if the chemotactic response of bacterial cells was triggered. Chemotaxis solution alone served as control. Each treatment was performed in triplicate.

In the capillary assay, a sealed disposable 200 µl pipette tip was used as the chemotaxis chamber for loading 100 µl of bacterial cell suspension. A disposable needle was used as the chemotaxis capillary and was attached to a 1 ml tuberculin syringe [B. Braun, Germany]. The needle-syringe capillary was filled with 100 µl of concentrated AREs of 7-day-old seedlings, or one of the two tested malic acid (dl-malic acid and l-malic acid), and tightly fitted into the sealed tips containing bacterial cell suspension. After 1 h of incubation at 28 °C, the content from each syringe was diluted 1000 times with liquid TSB and then plated onto TSA plates (3 plates for each chemo-attractant). The colony-forming units (CFUs) were determined by first plating on TSA plates and then incubated at 28 °C for 48 h. Chemotaxis solution alone served as control. Each treatment was performed in triplicate.

### Establishment of an in vitro agar system to investigate the interaction of *B. firmus* I-1582, *A. thaliana*, and *H. schachtii* at different pH levels

#### *B. firmus* I-1582 colonization at *A. thaliana* roots

Colonization by *B. firmus* I-1582 at *A. thaliana* roots was performed in a modified in vitro agar system as described below. First, 90-mm-diameter Petri dishes were filled with Knop medium (− sucrose) with pH 6.4, 7, 7.5 or 8, and then two droplets of Knop medium (+ sucrose) with pH 6.4 were added. Two sterilized *A. thaliana* seeds were placed on the sucrose-containing Knop droplets. The whole agar system was held at an angle of 60° to facilitate surface growth of the roots, and cultivated as described above. At 8 days post seeding, each seedling was inoculated with 10 µl of bacterial suspension (OD_600_ = 0.1) prepared from the 3-day-old bacterial culture. Seedlings inoculated with TSB alone served as control. After colonization for 12 days, a 1 cm root piece below the bacterial inoculation point was removed from the main roots and shaken vigorously in 1 ml of sterile water. A serial dilution of the suspension was plated on TSA. The CFUs were determined after incubating the plates for 48 h at 28 °C. Each treatment was performed in triplicate.

#### *A. thaliana* development colonized by *B. firmus* I-1582 living cells

10 µl of bacterial suspension (OD_600_ = 0.1) was inoculated to 8-day-old seedlings grown at pH 6.4, 7, 7.5 or 8 as described above. Seedlings inoculated with TSB alone served as control. After colonization for 12 days, aboveground-related parameters (shoot fresh weight and leaf number) were evaluated, underground-related parameters (root length, root surface and root tips) were measured using Epson Perfection V700 Photo scanner [Epson, Japan] equipped with WinRHIZO software. Each treatment was performed in triplicate.

### Impact of *B. firmus* I-1582 on *H. schachtii* parasitism at *A. thaliana*

#### Living bacterial cells (LBC), dead bacterial cells (DBC) and cell-free supernatant (CFS) preparation

To investigate the impact of *B. firmus* I-1582 on *H. schachtii* parasitism at *A. thaliana*, LBC, DBC, and CFS were applied separately. The main culture was cultivated at 28 °C for 72 h. LBC were pelleted by centrifugation at 4000 rpm for 10 min, washed by sterile water, and re-suspended in 50% TSB by adjusting OD_600_ to 0.1. DBC were subsequently produced by autoclaving at 121 °C for 20 min at 1.2 × 10^5^ Pa pressure. Bacterial supernatant was withdrawn and filtrated through a 0.2 µm filter. LBC, DBC and CFS were used freshly after preparation. As *B. firmus* is a spore-forming bacterium and as we only wanted to work with vegetative cells, we analysed whether spores could be found in the LBC preparation. Therefore, the cells were incubated at 80 °C for 10 min, a condition which spores survive but vegetative cells not. The heated cells and fresh LBC were spread onto TSA and incubated for 72 h at 28 °C. No colonies developed on the heated variant thus proving that the culture contains vegetative cells only.

#### Nematode infection and invasion assays

*A. thaliana* was grown on sucrose-free Knop plates with sucrose-containing Knop (pH 6.4) droplets as described above. At 8 days post seeding, each seedling was inoculated with 10 µl of prepared living cells or dead cells suspension (OD_600_ = 0.1). Seedlings inoculated with 50% TSB served as control. At 20 days post seeding, each plant was inoculated with 60–70 surface-sterilized *H. schachtii* J2s.

For the in vitro test system treated with CFS, two seeds were grown on the upper half plate filled with sucrose-containing Knop (pH 6.4). The plate was held at an angle of 60° and cultivated as described above. At 8 days post seeding, the CFS mixed with Knop medium was applied to the lower half plate. TSB mixed with Knop medium served as control. At 14 days post seeding, each plant was inoculated with 60–70 surface-sterilized *H. schachtii* J2s.

The infection assay was carried out to analyse nematode parasitism at *A. thaliana* plant by counting the number of males and females at 14 day-post-inoculation (dpi) under a Stereo Microscope [Leica, Germany], and measuring the size of female and syncytium at 28 dpi under a digital Stereo Microscope [Leica, Germany] equipped with Leica Application Suite (LAS) software. Each treatment was performed in triplicate.

Nematode invasion was quantified by evaluating the number and size of nematodes at 1, 2, and 3 dpi to examine the penetration ability of nematodes and their early development. Each treatment was performed in triplicate.

### Impact of *B. firmus* I-1582 on *H. schachtii* progeny at *A. thaliana*

To investigate the effect of *B. firmus* I-1582 on *H. schachtii* progeny, two assays were performed by using analogous experiment with LBC and TSB control as described above. First, cysts were collected at 35 dpi in 3 groups:

- ‘Ctrl’: cysts without bacteria colonization from TSB control plates,
- ‘w/o LBC’: cysts where colonization with *B. firmus* I-1582 is not visible under a microscope, and
- ‘w LBC’: cysts where colonization with *B. firmus* I-1582 is visible under a microscope.

The number of eggs of cysts of these 3 separate groups was evaluated for each individual plant. Each treatment was performed in triplicate.

In the second assay, the parasitism of juveniles that hatched from cysts of the described 3 groups was determined by inoculating *A. thaliana*. Therefore, cysts were collected separately at 84 dpi and incubated in 3 mM ZnCl_2_. After 7 days, 60–70 freshly hatched juveniles were inoculated to each 20-day-old plant. The invasion assay, infection assay, and reproduction assay were assessed at 1 dpi, at 14 dpi and 28 dpi, and at 35 dpi, respectively. Each treatment was performed in triplicate.

### Statistical analysis

All data are expressed as mean and median ± standard error (SE). Statistical analysis was performed by using Student’s *t*-test (p < 0.05 and p < 0.01) or one-way analysis of variance (ANOVA) (p < 0.05).

## Supplementary Information

Supplementary Figures.

## Data Availability

All data generated or analysed during this study are included in this published article and the supplementary material. More details are available from the corresponding author on reasonable request.
