# Supplementary material for: Bacillus firmus I-1582 promotes plant growth and impairs infection and development of the cyst nematode Heterodera schachtii over two generations
Source: Sci Rep. 2021 Jul 8;11:14114. doi: 10.1038/s41598-021-93567-0 (PMC8266893; doi:10.1038/s41598-021-93567-0)
Supplement: Supplementary file 1 — Supplementary Figures. [file 41598_2021_93567_MOESM1_ESM.pdf]

Supplementary Material

Bacillus firmus I-1582 promotes plant growth and impairs infection and development of the cyst nematode *Heterodera schachtii* over two generations

Mengmeng Huang[1], Aylin Bulut[1], Bidhya Shrestha[1], Christiane Matera[1], Florian M. W. Grundler[1] and A. Sylvia S. Schleker[1,*]

[1]INRES - Department of Molecular Phytomedicine, Rheinische Friedrich-Wilhelms-University of Bonn, D-53115 Bonn, Germany.

*Correspondence: sylvia.schleker@uni-bonn.de

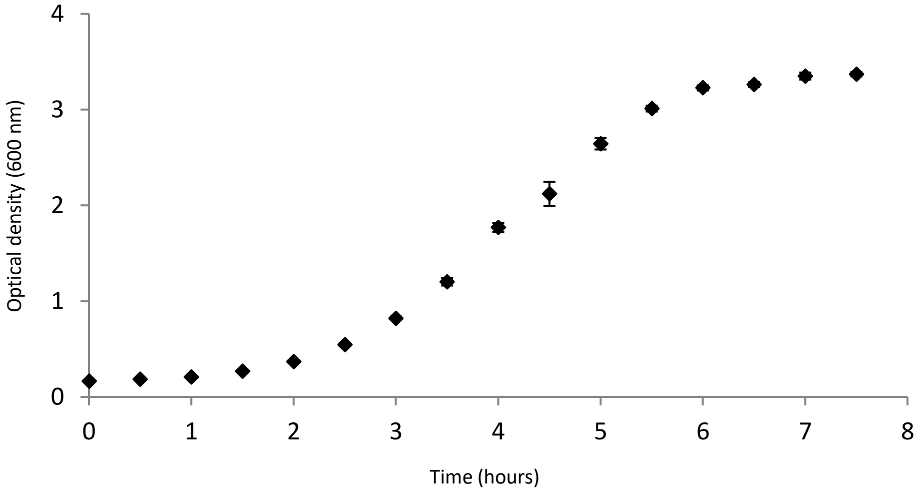

Supplementary Figure 1. The growth of *Bacillus firmus* I-1582 at 28 °C in TSB. Liquid bacterial culture was initiated with OD_{600} = 0.1. Results are expressed as the mean ± standard error of three independent biological replicates (n=6).

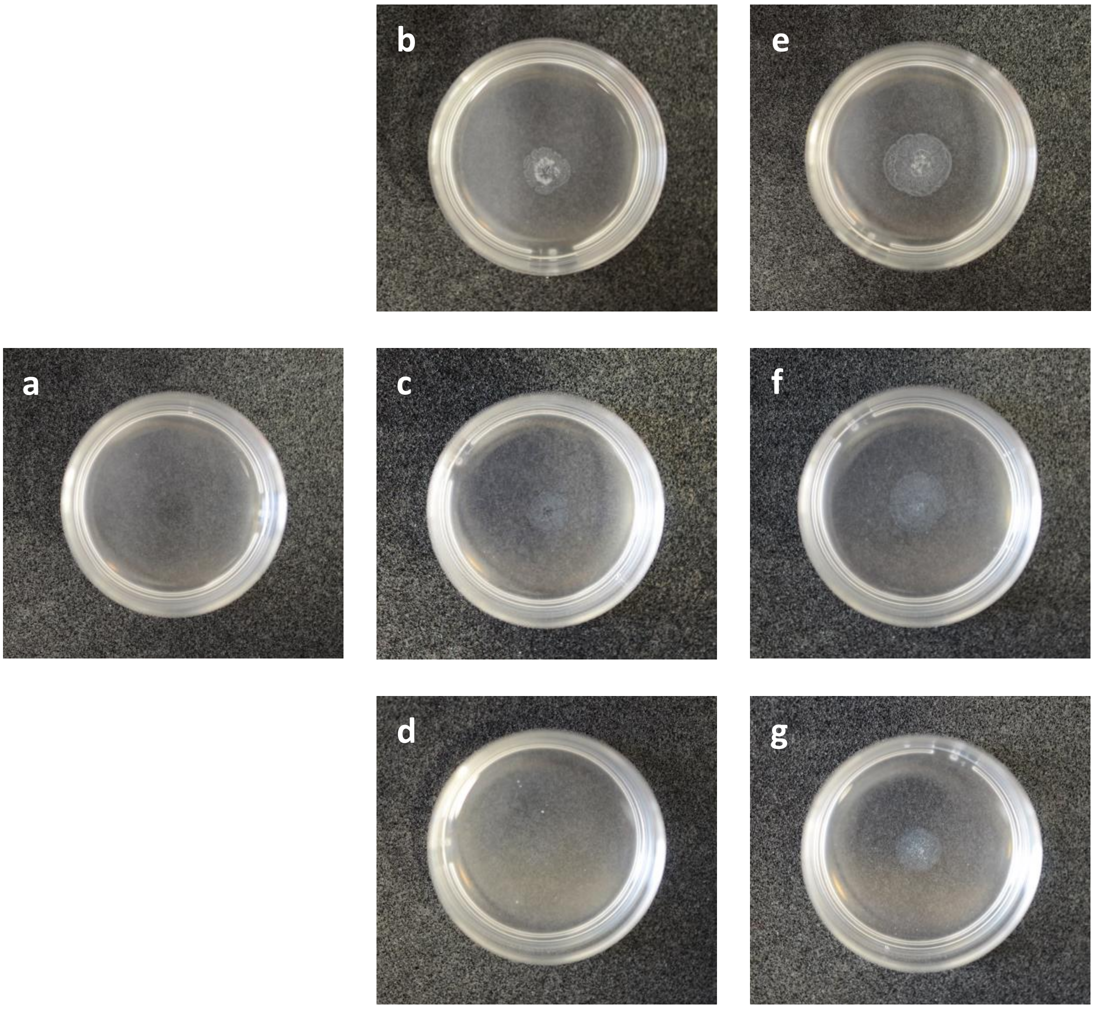

Supplementary Figure 2. Chemotactic response of *Bacillus firmus* I-1582 towards concentrated *Arabidopsis thaliana* root exudates (AREs) in a drop assay. A drop of the test substance was placed in the centre of a 35 mm diameter Petri dish containing *B. firmus* I-1582. A turbid ring indicated the aggregation of bacteria. 10 µl of chemotaxis buffer (**a**); 10 µl of AREs extracted from 7- (**b**), 21- (**c**), and 28- (**d**) day-old *A. thaliana* plants; 20 µl of AREs extracted from 7- (**e**), 21- (**f**), and 28- (**g**) day-old *A. thaliana* plants. Photographs are representative examples taken after incubation for 30 seconds at room temperature.

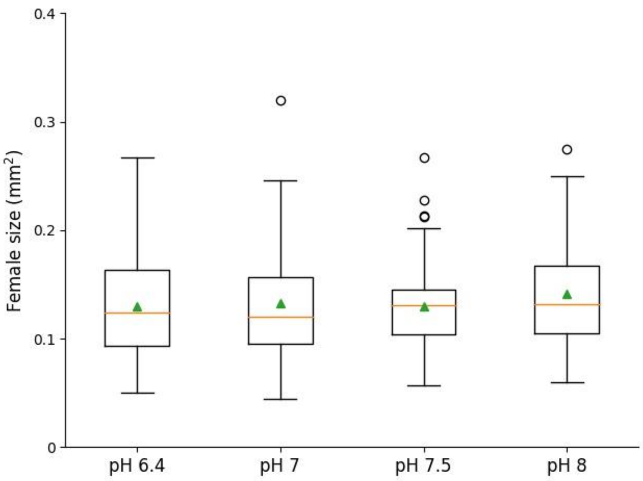

Supplementary Figure 3. Average female size of *Heterodera schachtii* at *Arabidopsis thaliana* roots at different pH at 14 dpi. Results are expressed as the mean (orange line) and median (green triangle) ± standard error of three independent biological replicates (n≥50). Data were statistically analysed using Dunn's Method. No significant differences were observed.

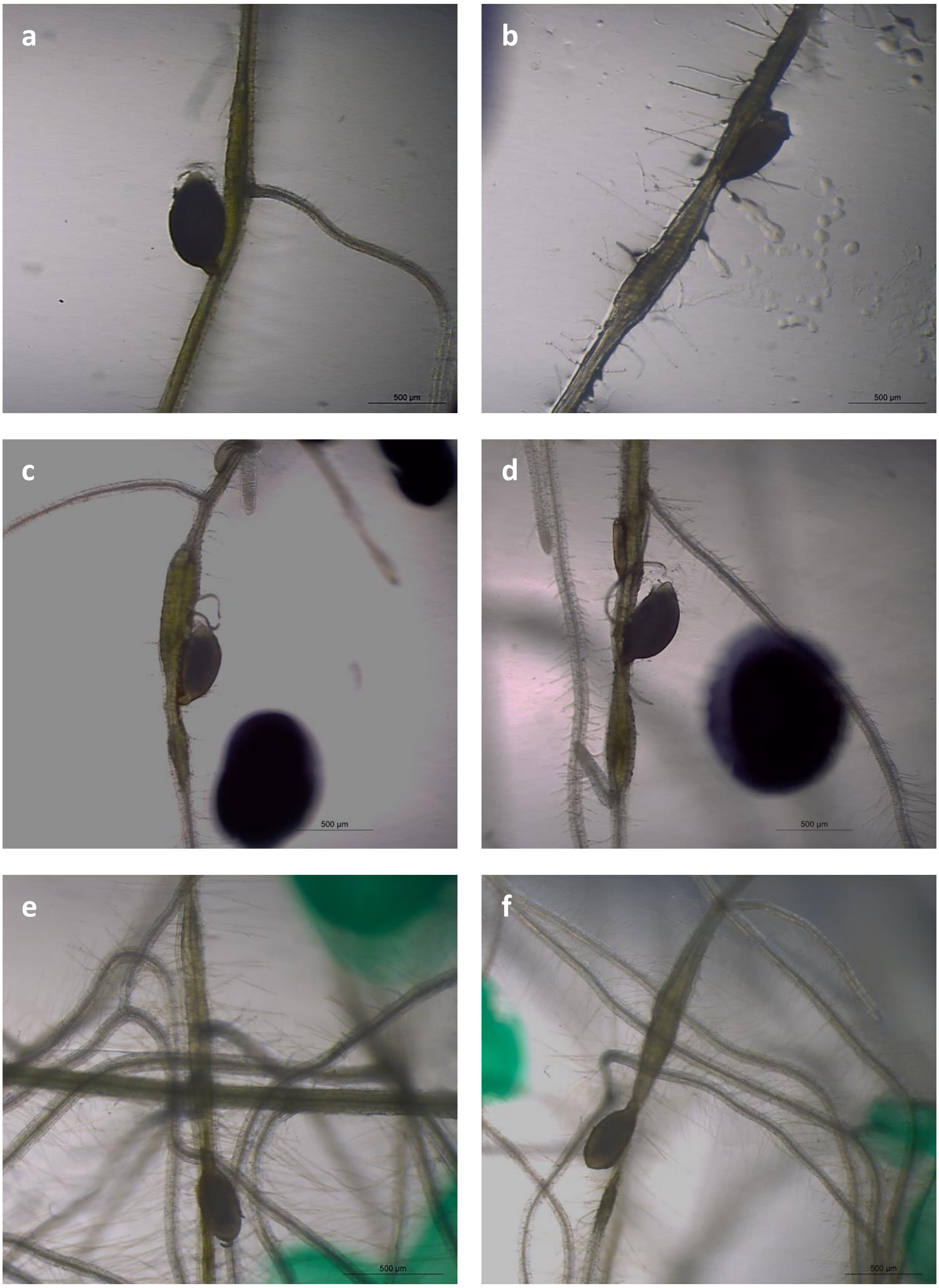

Supplementary Figure 4. Microscopic observation of females and syncytia with different treatments. Controls for living bacterial cells (LBC) (a), dead bacterial cells (DBC) (c), and cell-free supernatant (CFS) (e). Treatment with LBC (b), DBC (d), CFS (f). Photographs are representative examples taken 28 days post inoculation.

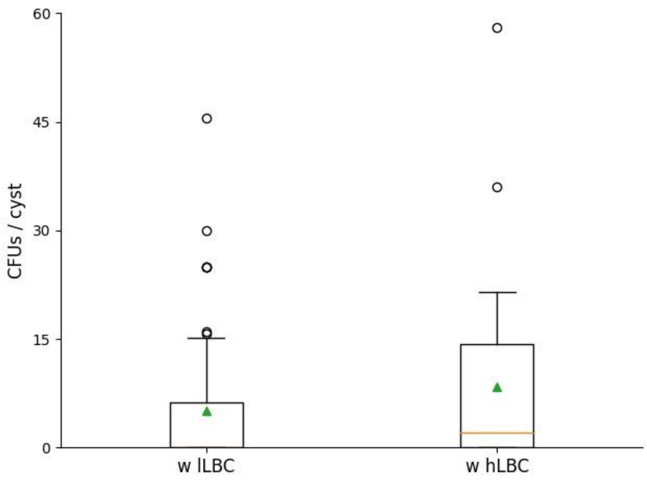

Supplementary Figure 5. Quantification of *Bacillus firmus* I-1582 attached to cysts of the 2nd generation. w hLBC: with high number of living bacterial cells; w lLBC: with low number of living bacterial cells. Results are expressed as the mean (orange line) and median (green triangle) ± standard error of three independent biological replicates (n≥42). Data are statistically analysed using Dunn's Method. No significant differences are observed.

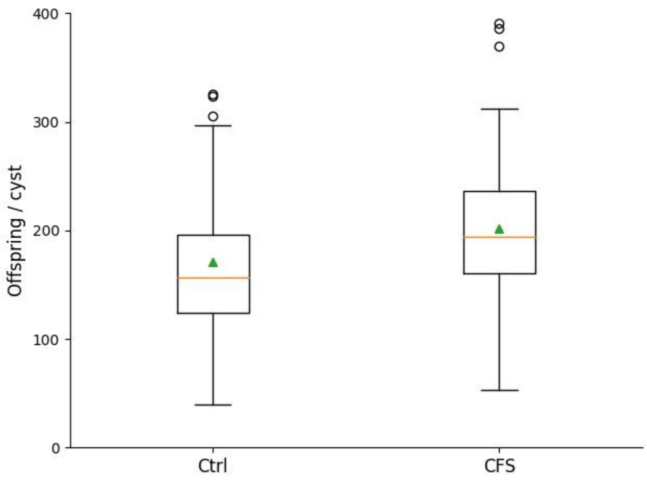

Supplementary Figure 6. Reproduction assay of *Heterodera schachtii* at *Arabidopsis thaliana* roots. Average number of eggs and juveniles per cyst at 35 dpi (days post inoculation) in the presence of CFS. Ctrl: control. Results are expressed as the mean (orange line) and median (green triangle) ± standard error of three independent biological replicates (n≥34). Different letters indicate statistically significant differences among treatments according to Dunn's Method (p<0.05).
